# Using group model building to frame the commercial determinants of dietary behaviour in adolescence – findings from online system mapping workshops with adolescents, policymakers and public health practitioners in the Southwest of England

**DOI:** 10.1186/s12889-025-21320-7

**Published:** 2025-01-14

**Authors:** Yanaina Chavez-Ugalde, Frank De Vocht, Russell Jago, Martin White, Zoi Toumpakari

**Affiliations:** 1 National Institute for Health Research (NIHR) School for Public Health Research (SPHR), Newcastle, UK; 2 Bristol Medical School, Population Health Sciences, University of Bristol, Bristol, UK; 3 NIHR Applied Research Collaboration West (NIHR ARC West), Bristol, UK; 4 Centre for Exercise, School for Policy Studies, Nutrition and Health Sciences, University of Bristol, Bristol, UK; 5 MRC Epidemiology Unit, University of Cambridge, Cambridge Biomedical Campus, Cambridge, CB2 0QQ UK

**Keywords:** Group Model Building, Adolescence, System mapping, Public health, Dietary behaviour, Commercial determinants of health

## Abstract

**Background:**

In England, 23% of children aged 11 start their teenage years living with obesity. An adolescent living with obesity is five times more likely to live with obesity in adult life. There is limited research and policy incorporating adolescents’ views on how they experience the commercial determinants of dietary behaviour and obesity, which misses an opportunity to improve services and policies that aim to influence the prevalence of childhood obesity. This study reports the findings from online Group Model Building system mapping workshops in which we explored the mechanisms by which commercial drivers influence adolescents’ dietary behaviour.

**Methods:**

We ran a series of 3 online Group Model Building workshops with adolescents and one Group Model Building workshop with policymakers and public health practitioners. Adolescents portrayed their views on how food and beverage industries influence what they choose to buy and eat in a system map, and then proposed a set of policy actions to promote healthier food environments. We shared the system map created by adolescents with policymakers and public health practitioners to reflect on how current policy interventions match adolescents’ views on the most influential factors.

**Results:**

The system map contains 37 elements connected by 70 hypothesised causal links and five feedback loops. These elements were grouped into six themes that portray the complexity of factors that influence adolescents’ food choices in their physical and digital environments, disproportionately encouraging the consumption of unhealthy products. Policymakers and public health practitioners reflected on the power and the deep level of influence food companies exert on adolescents’ behaviour. They recognised that the coexisting influence of food marketing and social media on mental health and body image is not well reflected in current policy and research efforts.

**Conclusions:**

This study highlights the need for public health policymaking processes to provide youth with a space to voice influential elements and consequences, thereby co-creating policies and designing interventions to buffer risk factors and increase well-being in this critical transitional stage.

**Supplementary Information:**

The online version contains supplementary material available at 10.1186/s12889-025-21320-7.

## Strengths and limitations of this study

- Group model building was a feasible, engaging, enjoyable and cost-saving method to engage participants with system thinking tools and system dynamic concepts
- Adolescents developed a comprehensive system map that reflected their views on how the commercial food system influences their dietary behaviour.
- GMB was a feasible approach to involve adolescents in research and an effective starting point to give voice to their views and develop policies that reflect what matters to them.
- Diversity in recruitment was limited, and having participants from different ethnicities and socio-economic positions could have identified slightly different factors and identified unique factors that widen health inequalities.
- Policymakers and public health practitioners found the system map a useful tool to visualise factors that are commonly left out when designing interventions and that go beyond interventions that require high agency from individuals.
- The study's sample does not encompass the diversity of the general population, and thus the results may not be generalizable beyond the specific group of participants included.

## Background

Adolescent obesity puts youth at an increased risk of greater long-term psychological and physical health problems [1]. Obesity tracks into adulthood and a child living with obesity is five times more likely to live with obesity as an adult [2]. The causes of obesity are multifaceted and have different drivers at different levels, from an individual’s genetic predisposition, their beliefs, skills and health behaviours, and their interaction with the physical environment and the commercial and political drivers of diet and physical activity [3–5]. Strategies to tackle obesity should include systemic drivers that go beyond individual-level solutions [6, 7]. A recent study that collated data from 10 local authorities in England found that there is a mismatch between the perceived causes of obesity and where intervention efforts are placed. Specifically, most intervention efforts aim to change individual lifestyle factors via educational approaches as opposed to targeting the wider determinants of health which involve the environment, policies or infrastructure [8].

Commercial drivers and the influence of food marketing on dietary behaviour, particularly the ones targeting adolescents, promote the consumption of energy dense, nutrient poor food products, which can contribute to the prevalence of overweight and obesity [9, 10]. A recent systematic review [11] defined the commercial determinants of obesity as food industry strategies focused at producing, promoting and increasing the sales of obesogenic foods and beverages, sometimes at the expense of public health. These strategies are of particular importance since they appeal to adolescents’ increased autonomy and agency alongside their urge for belonging and uniqueness [12].

Adolescence is a transitional phase within the life course that involves increased autonomy, independence and decision-making capacity, as well as creation and integration of social norms which sets adolescents in a unique position to become involved in nutrition policy by providing their lived experience and recommendations to improve their food choice environments [13, 14]. Adolescents are different from younger children since they exercise a greater food choice over what and when they choose to buy and eat [15]. Demographic changes have also resulted in the current group of adolescents being the largest generation of 10–24 year-olds in human history [16]. The large size of this group has made adolescents a key food and beverage industry target to increase sales and profits [17].

Systems approaches have been advocated as a way of understanding the complexity of the many interacting factors driving unhealthy diets and obesity [18, 19]. Group model building (GMB) is a facilitated, participatory modelling method based on system dynamics [20], where stakeholders elicit their mental models of a given problem and create a shared understanding of a complex system [21] in the form of a causal loop diagram (CLD) (i.e. system map). GMB helps in generating qualitative hypotheses’ of the dynamics of a complex problem [20]. GMB also makes it possible to visualise potential leverage points in the system where interventions can be delivered, which can guide the development of policy responses [22, 23].

Research shows that young people tend to be systematically excluded from decision making [24], and there is limited research incorporating adolescents’ views on how they perceive and experience the commercial determinants of dietary behaviour and obesity [25]. Youth participation and incorporating their views into decision-making processes can positively affect their personal development and can improve services and policies that aim to target [26].

This qualitative study aims to develop a depiction of the commercial obesogenic food system in adolescents in the Southwest of England, to identify the key elements of influence and their interrelations. This will allow hypothesising causal pathways and find potential opportunities for effective policy interventions to reduce/prevent obesity in adolescence. This aim will be fulfilled through ‘systems mapping’ workshops, using GMB as the core methodology, with a range of stakeholders, including groups of adolescents, as well as policymakers and public health practitioners, in the Southwest of England.

## Methodology

### Participant recruitment

#### Covid-19 restrictions

This qualitative study was initially designed to be in-person across to reach 4 schools from different socio-economic status (SES) (4 workshops of 10 adolescents aged 16 to 18 in each school; n = 40 adolescents) and two groups of policymakers and public health practitioners in the Southwest of England (2 workshops of 7 participants each; n = 14 policymakers and public health practitioners). However, with the Covid-19 lockdown restrictions the system mapping workshops had to be re-designed and changed to an online format, limiting the participant number and diversity we aimed to achieve initially. The re-design of the workshops into an online format and the detailed methodology we undertook has been published elsewhere [27].

#### Online recruitment during the Covid-19 pandemic

With the Covid-19 restrictions we recruited participants for this study online from May 2020. Thirteen community youth groups with available email accounts, based in the Southwest of England were contacted via their institutional emails. Previous research suggests that having 10 to 20 participants in online discussions allows the facilitator to effectively encourage meaningful interactions among them [28]. Therefore, we aimed to target a study population of 14 adolescent boys and girls (16-to-18-years old at the time of the workshops). Community groups were emailed an information sheet about the study and contact details to get back in touch via email in case any of their youth members wanted to take part. Three youth groups responded (Bristol Young People’s Advisory Group (YPAG), Avon Scouts, and Knowle West Media Centre), nine did not respond and one did not take part because of lack of interest in participating from any of their community members. We purposively recruited participants that showed interest in the study, after reading the study invitation and information sheet that will was shared via the Young People’s Advisory Group (YPAG) NIHR ARC West and did snowballing sampling after that to reach the recruitment numbers (n = 14). We recruited from these groups adolescents between 16–18 years, living in the Southwest of England, with access to a stable Wi-Fi connection who were willing to participate in the 3 online sessions (2 individual sessions and 1 workshop). Participants were offered a £30 online voucher for participating partly or in all three online sessions. Signed consent forms were emailed back to YCU to allow adolescents to take part in the study.

Public health practitioners and policymakers were recruited through existing networks of contacts (i.e., University of Bristol, NIHR Applied Research Collaboration (ARC) West) and snowballing by asking interested participants to approach others who could be suitable. We aimed to recruit between 10 and 14 public health practitioners and policymakers for our online discussions, as suggested by previous research [28]. All the study information (information sheet, consent forms, online platform joining instructions) was sent via email. Signed consent forms were emailed back to YCU to allow public health practitioners and policymakers to take part in the study.

### Group model building methods

The detailed methodology has been described elsewhere [27]. In summary, we used GMB as a method to produce a system map, in the form of a causal loop diagram (CLD), of the commercial determinants of dietary behaviour and obesity. The main parts of a CLD are: 1) the contributing elements to the problem; 2) connections between these elements; and 3) feedback loops. The elements (also called factors or variables) thought to be the cause or consequence of a given problem. The connections between elements explain the relationship between them and are denoted by a polarity (+ or –, also represented by a continued or a dotted arrow respectively). A positive polarity represents that a change in a cause variable will produce a response in the outcome variable in the same direction. A negative polarity means that a change in the cause variable will produce a response in the outcome variable in the opposite direction. Feedback loops are the circular nature of cause and effect and show the interaction between the system variables and how these interactions influence the system. Feedback loops are described as reinforcing ( +): an action that creates a result producing more of the same action, resulting in continued growth or decline. For example, the more money you have in a bank account, the more interest it will generate, which will generate an increase of money in your bank account. Or balancing feedback loops (-): an action that creates a result which produces the opposite direction of the initial action, and can be considered resistance forces, and help in maintaining stability or limiting growth. For example, when the temperature in a room goes below (above) certain threshold, the thermostat begins to increase (decrease) the temperature of the room to keep it within the threshold. Feedback processes are considered to give rise to the population distribution of complex health behaviours [29, 30]. Therefore, identifying feedback loops can point into promising leverage points to target interventions and change the systems’ behaviour.

### Overview of GMB sessions, workshop and data analysis

We conducted two individual online sessions and one online system mapping workshop with adolescents via *BlueJeans*, an online video conference software. Session 1 was an individual session with each adolescent in which they were introduced to the problem using a system mapping approach (i.e., elements, connections with polarity and feedback loops) and created their first system map. A software called *STICK-E* (Systems Thinking In Community Knowledge Exchange), which was developed in Deakin University, Melbourne, Australia, was used to visualise the system map [31]. Between session one and the GMB workshop, the system maps created by the adolescents were combined by YCU into one overarching system map containing all proposed elements and connections. We used this overarching system map to prompt discussion in the GMB workshop, which was held as a group. Supplementary material 1 provides a diagram with an overview of the two online sessions and the workshop.

In the GMB workshop, adolescents further developed the overarching system map, prioritised variables, explained causal connections between variables, and identified feedback loops. Adolescents were encouraged to examine the structure of the system map and change, add, or correct any misrepresentation of elements and feedback loops in the model. Once they agreed that the map correctly represented their thoughts, we considered the session concluded. The GMB workshop was video-recorded. Between the GMB workshop and the second individual session, we analysed the system map alongside the recording and notes taken during the workshop. Data analysis followed principles of thematic analysis and similar elements from the map were grouped into themes.

In the second online individual session, we shared the system map with each adolescent individually. They were asked to review and consider whether the map represented their ideas and how they meant them to be represented. Key feedback loops were articulated, and we refined narratives and themes. Once they agreed that the system map represented their thoughts accurately, the map was considered validated. The adolescents had no disagreement or non-validation during the group or individual workshops.

During the second individual session, adolescents were asked to devise solutions and generate a list of interventions or policies to target feedback loops that they considered important in developing the current system’s behaviour (i.e., one favouring unhealthy eating behaviour). Prompting them with solutions could have tainted their thoughts on policy solutions, so they were given enough time to think freely about ideas to target the system.

In a subsequent 1-h online workshop, the validated system map was shared with public health practitioners and policymakers. They were asked if they thought of any missing elements on the map that they considered important drivers to understand the commercial determinant of dietary behaviour in adolescence. They were prompted to have a wider discussion on the complexity of the commercial food environment and reflect on the power of big corporations, which prioritise profits, versus public health, which prioritises population health. Adolescents’ policy ideas were also shared with them to identify areas of current and unexplored policy intervention areas, and to discuss potential implementation of policy ideas. This session was video recorded in *BlueJeans.*

At the end of the adolescents’ and practitioners’ workshops they were asked to fill out an anonymous evaluation form to assess their experience of taking part in the GMB workshops. Results from the evaluation are reported elsewhere [27].

The research was approved by the University of Bristol Faculty of Health Sciences Research Ethics Committee (Ref: 99,003) and written informed consent was received from all participants. Participant data management and storage followed University of Bristol Information Governance principles. Personal data was saved in a password protected file and saved in the University of Bristol server. This research only shares anonymised data and participants are not personally identifiable.

## Results

### Participant characteristics

We recruited 11 adolescents. Table 1 shows the descriptive characteristics of adolescents.

**Table 1:** Descriptive table for adolescents' demographic characteristics

		**N (%)**
Gender	Female	8 (73)
	Male	3 (27)
Age	16	4 (36)
	17	6 (55)
	18	1 (9)
Ethnicity	White	11 (100)
Parental/carer highest educational attainment	University (Bachelor’s Degree or college of further education)	5 (45)
	Master’s degree	5 (45)
	PhD	1 (9)
Household employment status (parent/carer 1)	Employed full-time	10 (91)
	Self-employed	1 (9)
Household employment status (parent/carer 2)	Employed full-time	3 (27)
	Employed part-time	7 (64)
Currently receiving free school meals	No	11 (100)
Youth group	Bristol Young People’s Advisory Group (YPAG)	10 (91)
	Avon Scouts	1 (9)

Eight were female, median age was 17 years old and all of them were white. All adolescents had a parent/carer with a university degree, and five had another parent with a bachelor’s degree or college of further education, five had a parent with a master’s degree and one parent had a PhD. Ten adolescents’ households had at least one parent employed full-time and one had at least one parent self-employed. None of the adolescents received free school meals. In summary, the characteristics of the small sample we were able to recruit were mainly female and from a middle-high socioeconomic status.

We reached sixteen practitioners and policymakers. Table 2 shows policymakers and public health practitioner roles.

**Table 2:** Policymakers and public health practitioner roles

Policymaker/public health practitioner	Role
Policymaker 1	Clinical Commissioning Group in Southwest England
Policymaker 2	Local Authority in Southwest England
Policymaker 3	Local Authority in Southwest England
Policymaker 4	Local Authority in Southwest England
Academia and public health practitioner 1	Senior Research Associate in Southwest England
Academia and public health practitioner 2	Obesity Specialist Dietitian and Research Fellow in Southwest England

Four policymakers and two public health practitioners were recruited through existing network of contacts. Four (67%) were policymakers, whilst two (33%) divided their time between practice and academia.

### Results from the system mapping workshop

**Fig. 1:**
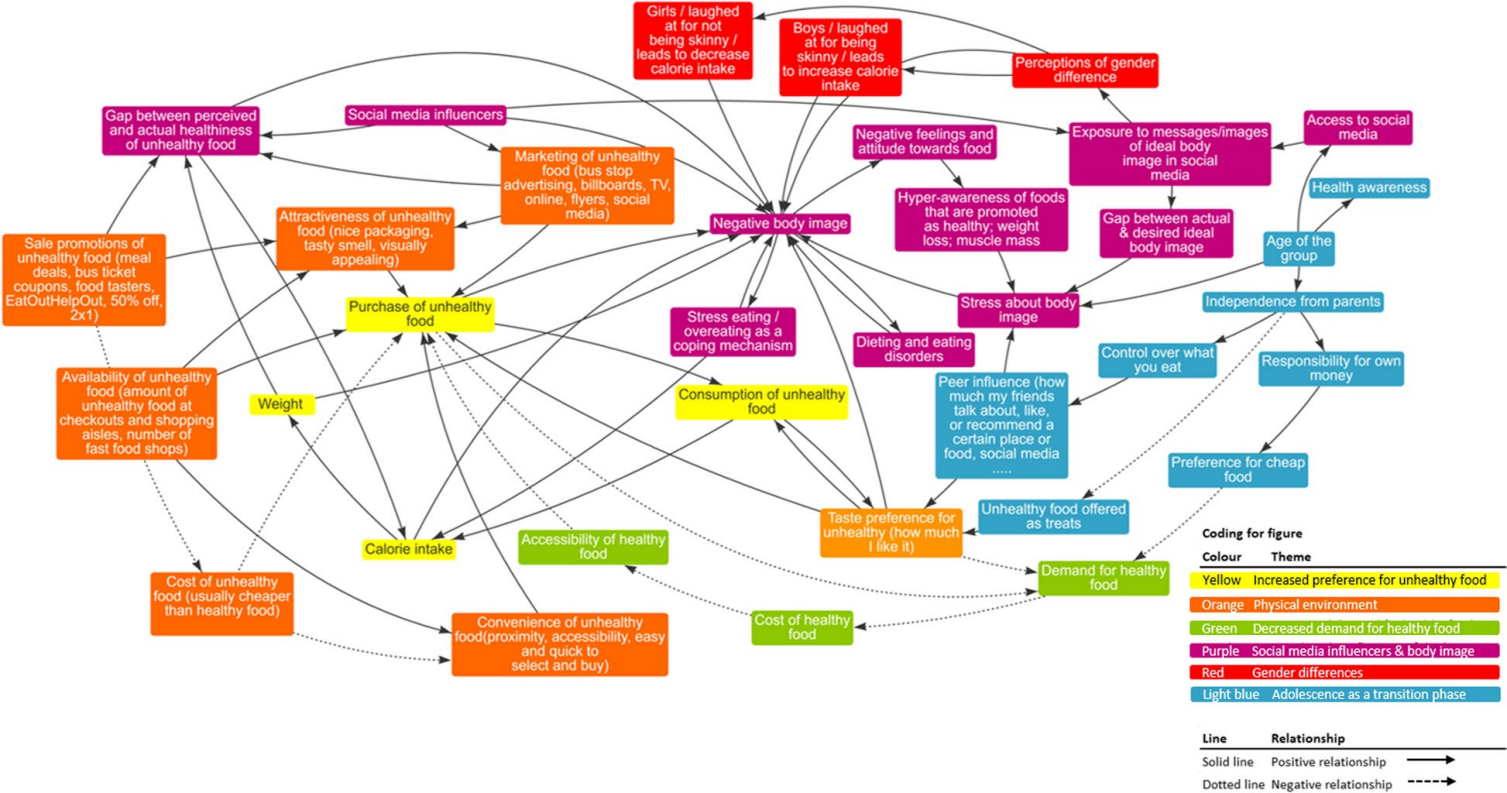
Validated system map created by the adolescents. Suggested instructions: This system map can be read in any direction. However, we suggest starting with the "Purchase of unhealthy food" box, highlighted in yellow, and following the yellow theme, which represents the most immediate factors affecting adolescents' weight status. Next, readers can proceed according to the themes/colours listed in the coding guidance at the bottom right of the figure. Finally, readers can explore the interconnections between factors by following the arrows across different themes in the system map

**Fig. 2:**
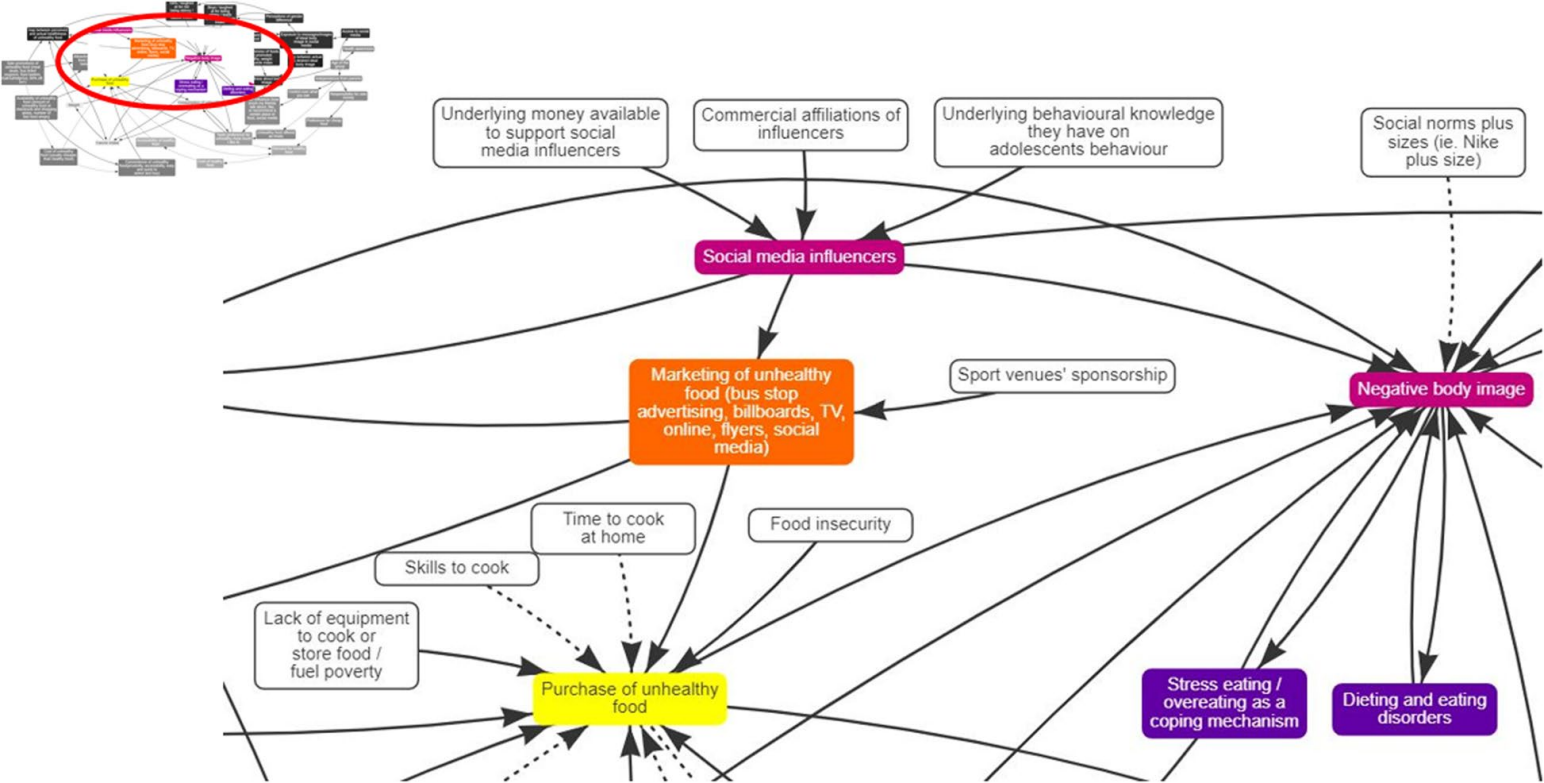
Additions to the system map by public health practitioners and policymakers. White boxes are the added elements by the practitioners and policymakers

Figure 1 shows the validated system map created by the adolescents and Fig. 2 shows policymakers’ and public health practitioners’ additions to the map.

### System map themes

The system map created by adolescents (Fig. 1) illustrates how adolescents perceive the commercial food system and how it drives their food choices. The system map contains 37 elements connected by 70 causal links and five feedback loops. These elements were grouped into six themes that are influenced by food and beverage corporations. The themes are represented by the different colours in the map and explained in detail below.

#### Theme 1: Increased preference for unhealthy food consumption and weight gain (yellow)

At the centre of the map, adolescents developed a behavioural pathway where they identified that an increased consumption of unhealthy food can lead to weight gain over time. Adolescents recognised that unhealthy food is usually high in calories, salt, fat and sugar, and that having an increased consumption of these foods, due to its high caloric content and taste appeal, can lead to weight gain over time. Additionally, they acknowledged that these foods are more abundant in their food environments, compared to healthy foods, increasing the likelihood that they will buy and consume these types of foods.

*“Unhealthy food is usually more attractive, convenient, available and cheaper than healthy food. This makes it more likely I will buy unhealthy food instead of healthy food. If I buy unhealthy food I will eat more of it and I will eat more calories, which will lead to more weight.” (Adolescent 1, female, 16)*

#### Theme 2: Physical environment that makes unhealthy food products abundant, highly marketed and attractive (orange)

Adolescents recognised that in their physical environment unhealthy foods are highly available, affordable, visible, attractive, convenient, and palatable leading to a taste preference for unhealthy food compared to healthy food.

*“I see a lot more marketing for things that are unhealthy, and a lot more effort into convincing people to buy those kinds of things.” (Adolescent 2, male, 18)*

An important comment they made, was the acknowledgement that marketing had an influence on how they perceived the foods and their purchasing behaviour. Even when they recognised a certain food to be unhealthy initially, marketing of these foods as being healthy prompted them to perceive them as “healthier” than they initially thought, increasing their intention to purchase them.

***“*** *An increased marketing of unhealthy food increases the perceived healthiness I have of that food, making it seem that it is healthier than it actually is through marketing it. So, marketing changes my perception of something making it more attractive for me to buy it.” (Adolescent 3, male, 17)*

Policymakers and public health practitioners also identified sport venues’ sponsorship as another form of marketing in adolescents’ physical environments (Fig. 5– 6).

*“I mean, most adolescents like sports and either see them on TV or go to see it live… and it is filled with marketing of burgers, pizza, sugary drinks, crisps.” (Academia and public health practitioner 1)*

One policymaker was working directly with local food businesses to create a healthier eating environment; however, they recognised the unbalanced way unhealthy food is being marketed and made available compared to healthy food.

*“I work a lot with food businesses in trying to get them to support the “Bristol Eating Better Award” offering healthier food and making them cheaper and more available. I am very conscious that the orange boxes here [physical environment’s influence] and thinking about the unbalanced marketing, attractiveness, the convenience, the availability, and the cost of unhealthy versus healthy food.” (Policymaker 2)*

#### Theme 3: Decreased demand for healthy food due to increased preference and purchasing of unhealthy food (green)

Participants noted the way in which unhealthy food is promoted, priced, and made available, which ripples through the system provoking a decrease in the demand for healthy food. Additionally, they perceive healthy food as being more expensive and less available than unhealthy food.

*“I see that healthy food are less marketed than unhealthy foods. And I would say that sales promotion, the marketing, the low cost, the convenience, the attractiveness, and the increased taste preference for unhealthy would increase how much unhealthy food I buy and will decrease my intention to buy healthy food.” (Adolescent 4, female, 16)*

#### Theme 4: Social media influencers, marketing of unhealthy products, and its effect on body image (purple)

Adolescents recognised that the effect of social media influencers is two-fold. On the one hand, social media marketing by influencers and celebrities has significantly increased over time, adding to the marketing of unhealthy food they experience in their physical environment and making unhealthy food ever-present in their physical and virtual environments. On the other hand, adolescents perceive influencers as having “ideal body types”. Therefore, an increase in social media influencers increases their exposure to messages and images of “ideal body types”, creating pressure between their actual and desired body type. This in turn increases stress about their body image, feelings of “negative body image”, potentially eating disorders, negative feelings towards food, and creates an increased awareness and demand for foods that are promoted as healthy (usually marketed by social media influencers).

*“If a celebrity that looks physically healthy and is constantly promoting a certain food, it increases my perception of how healthy I think that food is. If they look like that and promote that food, it makes me believe that I will also look like that. In social media now I see lot of foods that are being marketed as healthy and I am not so sure they are as healthy as they say. Still, I have desire to buy them, especially if my friends are buying it too.” (Adolescent 5, female, 17)*

*“As I grow older, I see more and more workouts and diets in social media. On Instagram I always see other girls promoting weight-loss drinks. It just stresses me out to see so much of it, and I question if I am doing enough.” (Adolescent 6, female, 16)*

Policymakers and public health practitioners’ identified elements that reflect a wider and much broader circle of influence where corporations exert their influence, and which are potentially “out of sight” from the adolescents’ experience (Fig. 2). For example, the often unseen and expansive commercial affiliations of social media influencers.

*“I think there is an increased impact of influencers in social media, and they usually have a commercial affiliation, and sometimes is quite opaque. The influencer may be talking about what they did during the day and talking about the cereal bar or whatever it is, and [adolescents] don’t even realise that (…) some of the stuff that people are saying has some sort of affiliation to a food company promoting not necessarily the healthiest of food. And I think it is something that you don’t realise even as an adult, let alone as an adolescent.” (Policymaker 1)*

They also highlighted the importance of the underlying money available to support social media influencers to market their products. In the current context of social media influencers, food companies are spending significant resources and time in trying to understand and influence adolescents’ behaviour.

*“(…) the budgets that are available to corporate organisations to essentially give that money to influencers is something quite big. I think because they (…) have a very good understanding of the way in which young people think, the places where they hang out, from a social media perspective, and they will spend a heck of a lot of money in trying to understand the behaviours and things that young people do and like. And I think this is always the case from a public health point of view, that the budgets aren’t necessarily all the same so there isn’t opportunity to really explore young people’s habits because the budgets just can’t compare.” (Academia and public health practitioner 2)*

They added that because of the deep underlying knowledge commercial brands have on adolescents’ behavioural patterns, social media and gaming platforms are being used as a very powerful marketing medium to target adolescents.

*“I just saw an example of this, so FIFA and Burger King identified teams that young people were more likely to play with in the virtual gaming world. Burger King had become the main sponsor for this team that aren’t very well known, but it was the team that everybody played with on FIFA, so Burger King had a very good understanding of young people and how they behave on FIFA’s online gaming world in a way they might be able to influence them on the online social/gaming world, and (…) through the knowledge they acquire they are able to accurately identify where they can have an influence upon.” (Academia and public health practitioner 2)*

In terms of factors affecting negative body image, they identified a marketing campaign from a known sports-clothing line to create a social norm around plus sizes, reducing the pressure for skinnier body types. However, they recognised that it could perpetuate ideals of certain “body types” and unintendedly normalise unhealthy weight.

#### Theme 5: Perception of gender differences (red)

Adolescents expressed that the norm for an “ideal body type” differs between boys and girls. Boys mentioned that they were expected to look strong, and that they were laughed at when they looked skinny. Girls mentioned they were expected to look fit and skinny, which can trigger behaviours like counting calories and dieting. They mentioned that these perceived gender differences affect and are affected by the previous theme (social media influencers, marketing of unhealthy products, and its effect on body image). They mentioned that through social media they are constantly exposed to images of “ideal body types” creating a standard for how they should look like. When there is a discrepancy between the ideal and real body type it increases a “negative body image” of themselves, stressing them about their body image, potentially leading to eating disorders (i.e., stress eating/overeating, or dieting) as a coping mechanism.

*“We [boys] are expected to be strong and have big muscles. Social media influencers on Instagram have amazing bodies and sometimes promote protein bars and muscle bulking powders. If I am going to look like that, I’ll go get some of that.” (Adolescent 7, male, 17)*

*“I’m lucky I am skinny. I like sports, I’m always on the move. A lot of my friends don’t look like me and I’ve seen how they stress about counting calories and losing weight.” (Adolescent 5, female, 17)*

#### Theme 6: Adolescence as a key transition age for targeted marketing strategies (light blue)

Adolescents recognised that as they age, they become more independent from parents, peer-influence becomes more important, they become more aware of their own health, have more access to social media, they have more agency over how and where they spend their money, and they have more choice and control over what they choose to buy and eat. This transitional phase, where autonomy and decision-making capacity grows, alongside their urge for belonging and independence, makes them a strategic age-group for food and drink companies’ targeted marketing and sales strategies that aim to create a lifelong loyalty for their products.

*“As you get older you get more control over the food that you do eat, like when you are younger you eat whatever your parents give you. However, as you get older you also get more influenced by the people around you. So, say, what your friends are eating, or what people in social media are eating. You might feel inclined to eating the same way.” (Adolescent 8, female, 17)*

### System map validation

After the adolescents validated the system map through internal consensus, the map was validated and checked against a recent systematic review that conceptualises the commercial determinants of diet associated with obesity [11] to support external validity (see Table 3) and identify factors or mechanisms identified by adolescents themselves which have not previously been mentioned in the literature.

**Table 3:** Comparison between the academic literature on the commercial determinants of diet and obesity [11] and factors identified by the adolescents during the GMB workshop

Systematic review results		GMB workshop results	
**Food corporations’ spheres of action**	**Corporate strategies**	**System map variable**	**Adolescent’s factors not mentioned in systematic review**
1) Political and legal	1.1) Framing the evidence and debate	• Gap between perceived and actual healthiness of nutrient poor food	• Gap between actual and desired body image • Negative body image • Eating disorders • Negative feelings and attitude towards food
	1.2) Influencing governance of food production, trade and investment towards a more liberalised trading environment globally	Not portrayed on the system map	
	1.3) Influencing policymaking process	Not portrayed on the system map	
	1.4) Limiting corporate liability	Not portrayed on the system map	
2) Production, processing and design	2.1) Reducing processing/manufacturing costs	• Cost of unhealthy food (cheaper than healthy food)	
	2.2) Increasing market share	• Availability of unhealthy food	
	2.3) Agribusiness food/ ingredient supply	Not portrayed on the system map	
3) Marketing and preference shaping	3.1) Promotion to increase brand awareness and visibility	• Sale promotions of unhealthy food • Availability of unhealthy food • Cost of unhealthy food • Convenience of unhealthy food • Marketing of unhealthy food • Social media influencers	
	3.2) Influencing consumers perceptions of product (real vs aspiration)	• Gap between perceived and actual healthiness of unhealthy food • Attractiveness of unhealthy food	
	3.3) Creating brand loyalty	• Key age group to influence purchasing behaviour • Targeting increased independence, autonomy and peer influence • Marketing targeting different genders • Developed taste preference for unhealthy food • Sale promotions of unhealthy food • Social media influencers	
	3.4) Product placement and distribution	• Convenience of unhealthy food • Availability of unhealthy food	
	3.5) Pricing	• Cost of unhealthy food • Sale promotions of unhealthy food • Lower demand for healthy food • Increased price of healthy food vs unhealthy food	

### Additional views by public health practitioners and policymakers

During Covid-19 lockdown public health practitioners and policymakers became aware of the increased time adolescents spent on social media and how marketing, alongside adolescents’ increasing independence, influenced where they chose to buy their food.

*“I have two adolescent children, and I have seen such a shift, particularly during [Covid-19] lockdown, where they have had much more time spent on social media over this period of time. (…) adolescents’ getting more independence from parents and having more control over their food they are going for the cheap option. They don’t want to spend time cooking, they want nicely presented, easy to eat, cheap food.” (Policymaker 2)*

Furthermore, policymakers and public health practitioners highlighted factors related to health inequities and identified social determinants that increase the purchase of unhealthy foods. For example, the lack of equipment, time and skills to cook, and food insecurity, are elements that increase the purchase of unhealthy food due to its cost, convenience, and availability.

*“It is not a lack of willpower, some people just do not have the skill to cook, or the time to cook fresh food at home. (…) a meal deal, you just grab it on-the-go and get on with your day, you don’t even have to think about it.” (Academic and public health practitioner 2)*

Finally, they emphasised that having adolescents’ views on what influences what they choose to buy and eat is essential to understand what has been left out in research and policymaking efforts and to develop relevant policy options for this age group.

### Opportunities for interventions to improve adolescents’ food environments

**Fig. 3:**
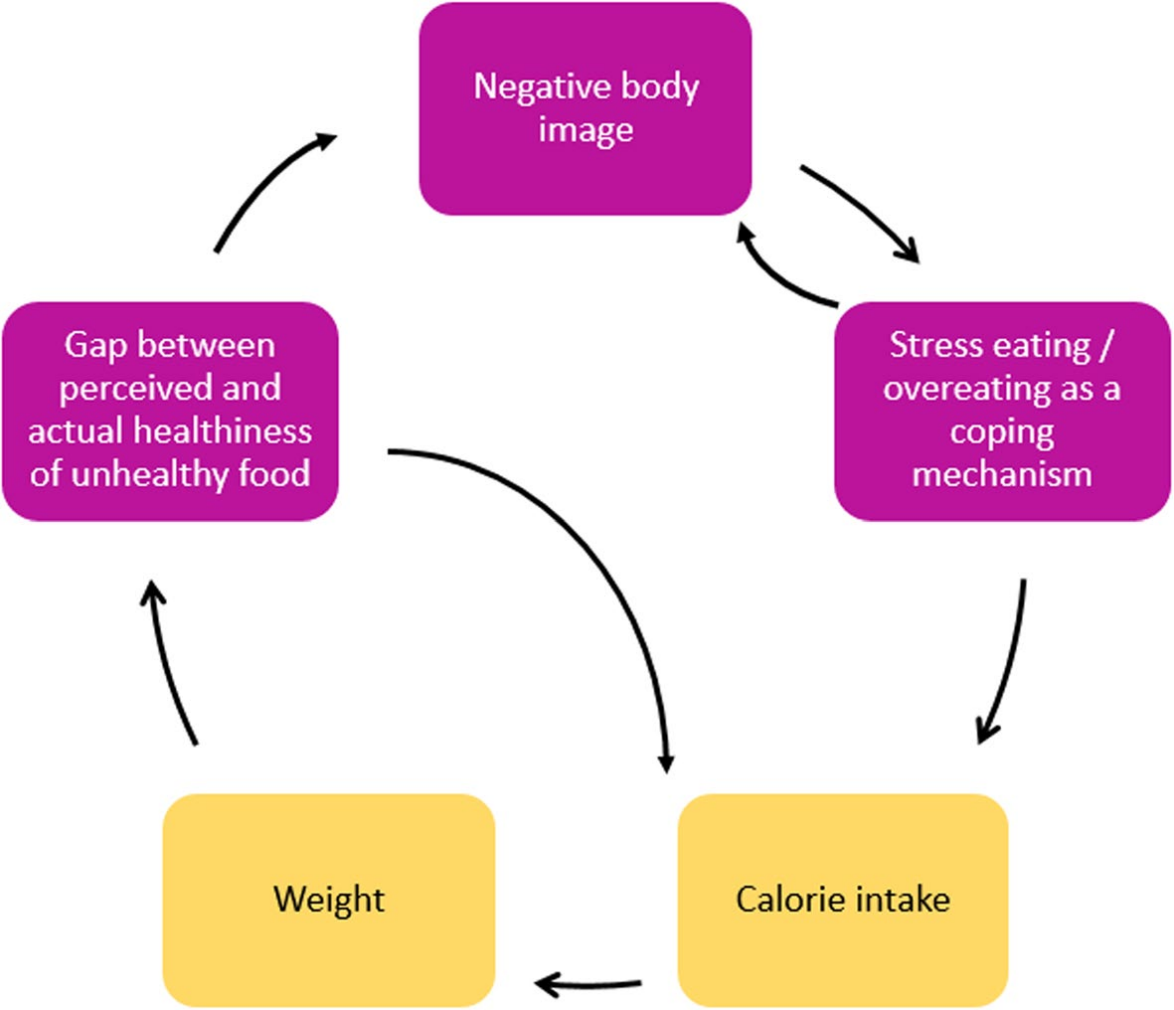
Feedback loop 1 and 2: Gap between perceived and actual healthiness of unhealthy food and negative body image

The system map includes five feedback loops, which identified potential leverage points for interventions to improve adolescents’ food environments (Fig. 3 to Fig. 6). Table 4 shows the potential interventions identified by adolescents, the themes that they could influence, and public health practitioners and policymakers’ comments on the interventions adolescents suggested.

**Table 4:** Policy ideas developed by adolescents

Policy / intervention	Themes they could influence	Feedback loops they could influence	Comments about potential implementation of suggested interventions by policymakers	Overall comments from policymakers and public health practitioners’
Regulating social media influencers that promote unhealthy foods masked as healthy	Social media influencers, marketing of unhealthy products, and its effect on body image	Feedback loop 1 and 2: Gap between perceived and actual healthiness of unhealthy food and negative body image	Outside their local authority remit	• They reflected on the unparalleled power some food companies have compared to public health and highlighted potential governance issues to implement certain policies • They discussed the importance of having a better understanding of adolescents’ behaviour and improve the design, framing and implementation of food policy for this age group to meaningfully influence their behaviour • Policies must tap into what young people care about. For example, focusing on the environment and climate change, like eating less processed food, can be a promising in helping with healthier diets but without focusing on calories or stigmatising weight status
Introducing warning labels on unhealthy foods	An increased preference to unhealthy food consumption and weight gain	Feedback loop 4: Food preference shaped by continued consumption of unhealthy food	No comment	
Reflecting the real long-term cost (of unhealthy food by making it more expensive and subsidising healthy food and making it more accessible to become the easy option	A decreased demand for healthy food due to increased preference and purchasing of unhealthy food	Feedback loop 3: Food demand which favours purchase of unhealthy food	One policymaker is working directly with local food businesses to create a healthier eating environment by offering healthier food and making them cheaper and more available	
	An increased preference to unhealthy food consumption and weight gain;	Feedback loop 4: Food preference shaped by continued consumption of unhealthy food		
Reducing/eliminating additives and flavour enhancers in unhealthy food	An increased preference to unhealthy food consumption and weight gain	Feedback loop 4: Food preference shaped by continued consumption of unhealthy food	Outside their local authority remit	
Regulate social media posts and encourage social media influencers and food companies to portray different body types in marketing and social media, and avoid celebrities, with idealised body types, promoting unhealthy foods	Social media influencers, marketing of unhealthy products, and its effect on body image	Feedback loop 5: Exposure to images of "ideal" body types leading to negative body image	Outside their local authority remit	
	Perceptions of gender differences	Feedback loop 1 and 2: Gap between perceived and actual healthiness of unhealthy food and negative body image		

#### Feedback loop 1 and 2: Reducing the gap between the perceived and actual healthiness of foods

Figure 3 depicts adolescents’ views on how social media influencers and the excessive marketing of unhealthy food create a gap between the perceived and actual healthiness of food. Participants suggested that some foods are promoted as healthy and perceived to be healthier than they actually are. This creates an increased gap between the perceived and the actual healthiness of food leading to an unintended increase in calorie intake, and over time, leading to weight gain.

This feedback loop connects to a larger feedback pathway that connects the gap between perceived and actual healthiness of food and negative body image, which leads to overeating as a coping mechanism, increased calorie intake and weight gain over time.

Adolescents suggested that reducing the gap between the perceived and actual healthiness of foods, could be a promising intervention and not only influence calorie intake but also the negative body image and overeating as a coping mechanism. To tackle this, adolescents suggested policies to regulate social media influencers that promote unhealthy foods masked as healthy and introducing warning labels on unhealthy foods.

Policymakers and public health practitioners were interested in listening to adolescents’ ideas to create healthier food environments and additionally highlighted potential governance issues since this would lie outside their local authority remit.

*“They came with many great policy ideas, but many of them are outside local control in a way. It is subject to big food industry branding and particularly when it comes to who are the big players with the money to promote and advertise, they tend to be the ones that are broadly unhealthy offers that they have.” (Policymaker 1)*

#### Feedback loop 3: Increasing the demand for healthy food

**Fig. 4:**
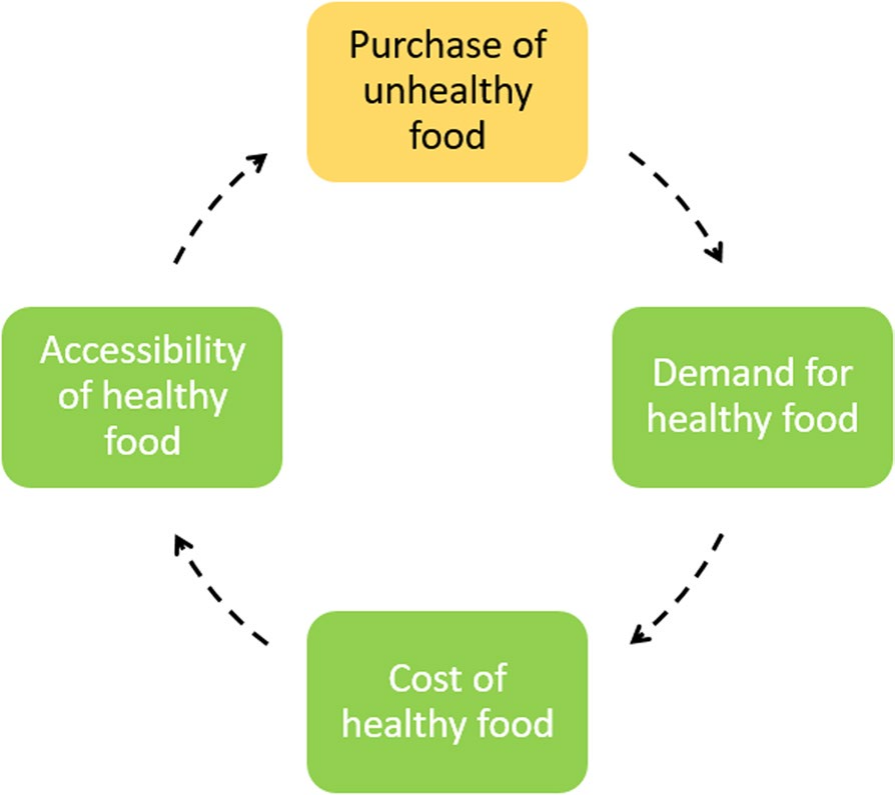
Feedback loop 3: Food demand which favours purchase of unhealthy food

Figure 4 illustrates adolescents’ reflections on how food companies make unhealthy food increasingly available, convenient, and attractive through marketing, compared to healthy food [32]. This is the result of the drive to make their profit margins larger by increasing the demand of their products and making more people buy more of their products through the aforementioned factors (i.e. increased availability, convenience, visibility). This leads to increased purchasing of unhealthy food and decrease the demand for healthy food, while at the same time increases the cost of healthy food, and decrease the accessibility of healthy food.

It was suggested that a potential new intervention / strategy to increase the demand of healthy food was to reflect the real long-term costs of unhealthy food (i.e. externalities, such as obesity, NCDs, and climate change) by making it more expensive, and subsidising healthy food and making it more accessible.

One policymaker was working directly with local food businesses to create a healthier eating environment by offering healthier food and making them cheaper, more available and visible within their shops. They felt that increasing the demand for healthy food could be partly addressed locally, for example by reducing advertising of unhealthy foods, and subsidising healthy food like fruit and vegetables.

#### Feedback loop 4: Reducing taste preferences for unhealthy food

**Fig. 5:**
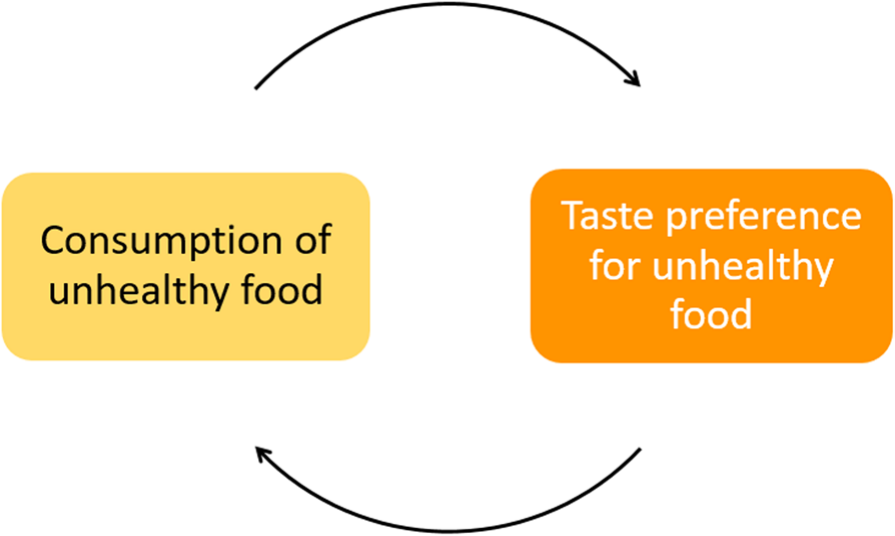
Feedback loop 4: Food preference shaped by continued consumption of unhealthy food

Figure 5 shows adolescents’ perceptions on how an increased consumption of unhealthy food creates a taste preference for unhealthy food, and they recognised how peer influence and being offered unhealthy foods as treats shaped their food preference from an early age.

Adolescents suggested reducing/eliminating additives and flavour enhancers in unhealthy food to reduce the taste preference for unhealthy foods, therefore, reducing the consumption of unhealthy food.

Policymakers commented that regulating powerful food companies’ product ingredients or formulations would be outside local policy remit. However, they highlighted that the soft-drink industry levy has had some influence on this since it has provoked reformulations from industry (i.e., reducing sugar content in sugar-sweetened beverages).

#### Feedback loop 5: Regulating social media posts and encouraging portrayal of different body types

**Fig. 6:**
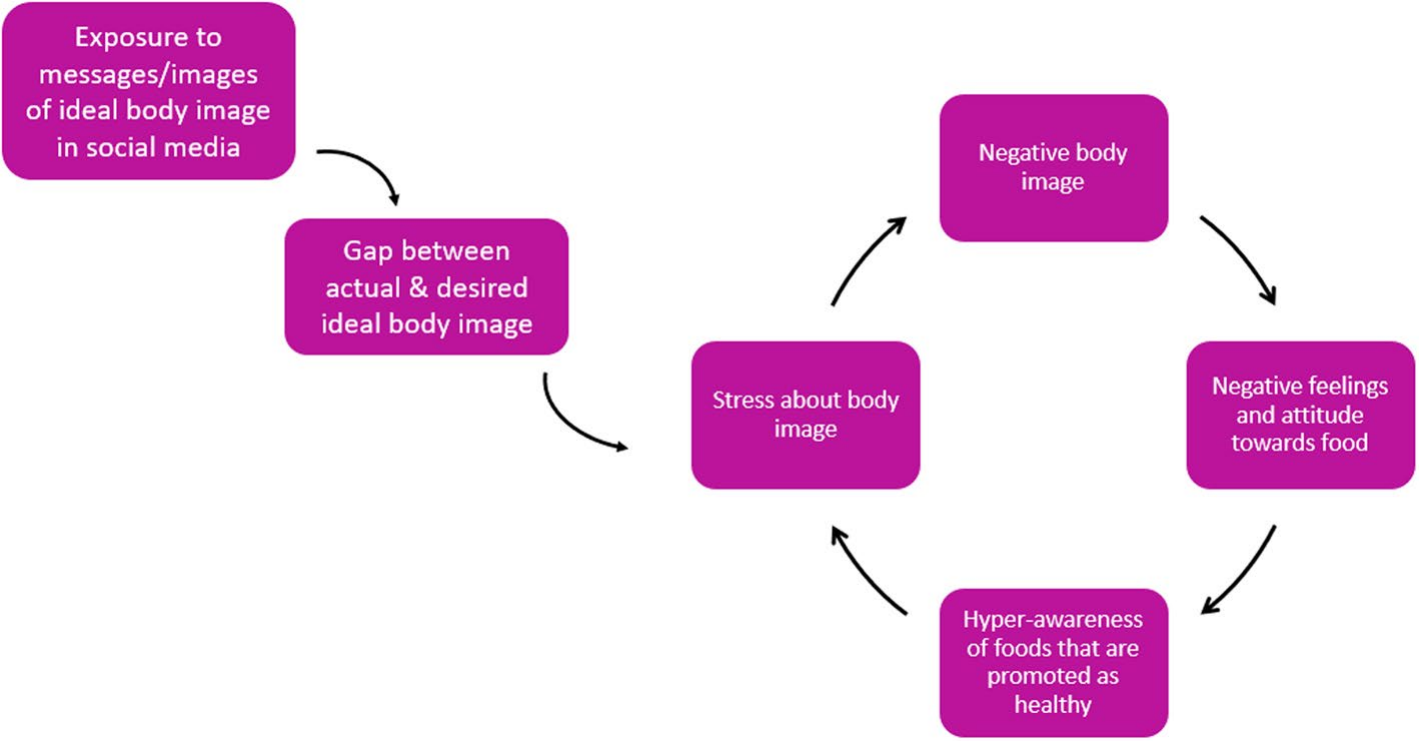
Feedback loop 5: Exposure to images of "ideal" body types leading to negative body image

Figure 6 illustrates how social media influencers increase adolescents’ exposure to messages of “ideal body types” and creates a gap between their actual and the idealised body type. This feeds into a feedback loop that increases stress about body image, increased negative feelings and attitude towards food and an increased awareness of foods that are promoted as healthy. Additionally, negative body image can lead to unhealthy eating patterns such as compulsive eating or eating disorders as a coping mechanism to deal with stress about body image.

Social media and exposure to ideal body types were perceived to have an impact on adolescents’ body image perpetuating misleading standards of health and attractiveness. To counter this, adolescents suggested an intervention to regulate social media posts and encourage social media influencers and food companies to portray different body types in marketing and social media, and avoid celebrities, with idealised body types, promoting unhealthy foods.

Table 4 presents a summary of the policy/intervention ideas developed by the adolescents, the themes these interventions could influence, and policymakers and practitioners’ comments.

### Additional comments from public health practitioners and policymakers

Policymakers and public health practitioners reflected on the importance of having a better understanding of adolescents’ behaviour and improving the design, framing and implementation of food policy for this age group to really influence their behaviour.

*“As an adolescent the more you are told you can’t have something, the more you want it. So in the case of food labelling as good, or bad, healthy or unhealthy, is not very useful. (…) an impactful policy has to be based on understanding their relationship with food to target that.” (Policymaker 4)*

They also suggested that health policies must tap into what young people care about. For example, focusing on the environment and climate change, like eating less processed food, can be a promising in helping with healthier diets but without focusing on calories or stigmatising weight status.

*“With increased awareness among young people about the environment and climate change, I think that one of the ways in which we could possibly avoid the framing of health and obesity (…) perhaps divert the attention off the weight which is often times counterproductive and obviously can have other connotations like stigmatising weight status.” (Policymaker 2)*

## Discussion

In this study, we completed a series of GMB workshops where adolescents portrayed on a system map their views on food industries’ activities and strategies that have an influence on what they choose to buy and eat, and developed policy ideas to tackle these factors to create healthier food environments.

The system map presents six themes: 1) an increased preference to unhealthy food consumption and weight gain; 2) a physical environment that makes unhealthy food products abundant, highly marketed and attractive; 3) decreased demand for healthy food due to increased preference and purchasing of unhealthy food; 4) social media influencers, marketing of unhealthy products, and its effect on body image; 5) perceptions of gender differences; and 6) adolescence as a key transition age for targeted marketing strategies. By using a systems approach with adolescents we offer a novel tool to visualise the complex system of dietary behaviour in this commonly-excluded age group [14, 15] and contribute to change the linear and individual-level paradigm for obesity prevention [33–35] into models that include the individual, social, economic, commercial and political drivers of unhealthy diets and obesity.

This research exposed the role of social media influencers, adolescents’ increased independence, gender differences, body negativity, and pervasive marketing in their physical and online environments. When the system map was shared with policymakers and public health practitioners, they were impressed by the level of detail the adolescents were able to portray in the system map. Since there is limited research involving adolescents’ views and input into policymaking efforts [12, 23, 36], they recognised the value of involving adolescents in this kind of research because it provides novel and valuable insights into factors and interactions between factors that have the most influential effect on their behaviours. Most importantly, they reflected on the power and the deep level of influence food companies exert on adolescents’ behaviour and they recognised that the influential factors highlighted by the adolescents were not well reflected in current policy and research.

There is limited research involving adolescents and getting their views on how they experience the commercial influence on their dietary behaviour. A recent qualitative study explored the views of 257 adolescents from five European countries on the drivers of obesity using GMB [23]. Although the aim of the study was not focused on identifying commercial determinants of obesity, commercial influence was identified as an important driver of unhealthy diets. Like the findings of this study, social media use, body image, pervasive marketing of unhealthy food and the power of big food companies were highlighted as important drivers of unhealthy behaviours. This strengthens the evidence for the role commercial drivers and their influence on dietary behaviours associated with obesity.

Adolescents identified five feedback loops and related leverage points that highlighted opportunities to target interventions or policies and improve their food environments. They highlighted the importance of reducing the gap between perceived and actual healthiness of unhealthy food influenced by social media influencers and excessive marketing of unhealthy food. Previous research has suggested that the impact of social media influencers on adolescents’ dietary behaviours has different mechanisms. For example, exposure to and high engagement with food-related content promoted by social media influencers is linked to poorer diet quality among adolescents because of their increased consumption of foods high in fat, sugar, and salt (HFSS) [37]. Our study found that even when adolescents use social media for health purposes, influencers often promote foods with a "health halo"—foods perceived as healthy but are actually energy-dense and nutrient-poor. This creates confusion among adolescents about whether these products are truly healthy. Similarly, a systematic review found that while adolescents use social media to search for fitness and healthy food choices, there is no clear evidence that health claims in promotional content lead to healthier food choices [38].

Furthermore, adolescents mentioned that their exposure to images of "ideal" body types is linked to body negativity. Participants considered social media influencers as another source of marketing and promotion for unhealthy food, making unhealthy foods increasingly attractive and accessible, consequently decreasing the demand for healthy food. A recent review found that influencers often present unrealistic body images, promote unhealthy foods, and commonly give inaccurate health advice [39]. Furthermore, they are not health experts and their commercial interests represent challenges to safeguarding adolescents’ health. Adolescents emphasised the role of social media influencers (i.e., individuals providing endorsements and product placements in their social media accounts) have on their food choices and body image, providing novel insights into adolescents’ online experience and how it affects their behaviour. While influencers create a gap between the perceived and the actual healthiness of unhealthy foods by marketing these as healthy options, in contrast the increased exposure adolescents have to an idealistic prospect of what a beautiful body and a healthy weight should look like also creates pressure on their body image. The latter might lead to increased intake of unhealthy products, which are promoted as healthy, stress eating, or eating disorders.

Adolescents suggestedtargeting factors which make their current food environment favourable for the purchase of unhealthy food due to how they are unequally made convenient, attractive, marketed and available compared to healthy food; regulating addictive substances in unhealthy food (i.e., sugar, caffeine, flavour enhancers) to break the cycle of taste preference and increased consumption of unhealthy food. There is substantial evidence showing that food environments are often unhealthy and dominated by nutrient-poor, energy-dense foods that are widely accessible. For instance, fast food outlets are more common in certain urban neighbourhoods [40] and retail shops tend to give more shelf space to unhealthy food products compared to healthier options [41]. In our study, adolescents recognized how easy it is to access unhealthy foods and how challenging it is to find affordable, healthier options.

A key feature in this study is that even when the literature has identified some of the factors mentioned in this study, this study used a “systems approach” where adolescents moved away from a linear understanding of their dietary behaviour and provided a systemic and dynamic perspective (i.e., feedback loops) on how they experience the commercial determinants of dietary behaviour and obesity. The themes and five feedback loops identified by the adolescents could form the foundation of new intervention strategies.

Existing public health interventions that tackle unhealthy diets tend to be focused on individual-level changes rather than upstream actions required to alter the systemic drivers of behaviour [34]. Adolescents mentioned an intervention focused on individual-behaviours (i.e., warning labels), and one that focused on industry reformulation, similar to the implemented soft-drink industry levy in the UK [42]. However, they mostly focused on interventions that could alter the structural drivers of dietary behaviour with a much broader influence on body image, eating disorders and perceptions on gender differences. Adolescents were therefore focussed on the issues that could form the basis of more upstream interventions that might lead to system change. Addressing these issues will be challenging but the insights from this study may aid in shifting the policy paradigm from a focus on individual behaviours to a focus on the systemic drivers of unhealthy diets.

### Strengths and limitations

We believe this is the first study to use GMB workshops to understand how adolescents perceive the commercial food system and the influence it has on their dietary behaviours. GMB proved to be a feasible approach of involving adolescents in research and an effective starting point to give voice to their views and develop policies and interventions that reflect their experience. Policymakers and public health practitioners thought the session was very valuable. They mentioned that the system map was a useful tool to visualise factors that are usually left out when designing interventions and go beyond individual behaviours.

A limitation of this study was the reduced number and diversity of adolescent participants. Having a more diverse group, including participants from different ethnicities or socio-economic groups could identify slightly different factors in the map, it could explain identified pathways in various ways and enable exploring unique factors of influence in different subgroups and reduce social and health inequalities [43].

Participants were mainly girls, and research on dietary behaviours suggests that girls are prone to have a higher body negativity compared to boys, are more likely to over eat under the influence of emotions, and are more likely to apply dietary restrictions to themselves than boys [44]. Participants in this study were not screened for mental or eating disorders, potentially skewing the results, and reflecting participants’ biases. Another limitation was that due to COVID-19, the time available from public health practitioners and policymakers was restricted, and we could only have one 1-h session. A longer or subsequent session would have been beneficial to explore more in-depth their views on potential effective policy ideas and barriers to their implementation. Finally, as with any qualitative research, the results presented in this research are subject to participants views and may not be generalisable to the whole UK population since it only included people from the South West and those engaged through their various youth groups. Due to the non-representative nature of the sample, the generalizability of the study's conclusions to the wider population is limited.

### Policy implications

Incorporating systems thinking methods into policymaking has the potential to increase policy innovation and effectiveness, and reduce unintended consequences of good-intentioned practices from other contexts [45]. It can actively foster dialogue between diverse stakeholders and enlighten the mechanisms by which commercial drivers influence core dietary behaviour and affect health a long time into the future. Furthermore, incorporating systems thinking as a decision-making tool can foster shared understanding of a complex problem by portraying it in a system map that depicts causal pathways, feedback loops and network structures, and match intervention efforts to target different levels in the system to achieve systemic changes [18, 23, 46]. One policymaker expressed interest in taking this work further and discussing social media influencers as potential intervention points to encourage healthy eating among adolescents.

Future studies should further explore the opportunities for interventions that adolescents suggested as potential ways of improving the food environment. For example, regulating social media posts and encourage social media influencers and food companies to portray different body types in marketing and social media, and avoid celebrities, with idealised body types, promoting unhealthy foods. This reflects that adolescents identified upstream interventions that go beyond the local remit of the policymakers involved in this study, and stressing the importance of implementing obesity policy interventions at national level. Additionally, involving a more diverse group of adolescents in system mapping workshops to uncover commercial drivers of unhealthy diets can increase our understanding of potential health inequalities related to dietary behaviour. This qualitative system map (i.e., CLD) was created using a GMB technique, which is based on system dynamics methods. This system map could be used as a blueprint to translate this CLD into a system dynamics model, including stocks, flows, accumulations, and time delays, can expand into a quantitative simulation platform for policy analysis and test potential outcomes of the policy actions suggested by the adolescents.

## Conclusion

This study demonstrates the value of involving adolescents in understanding and addressing the commercial determinants of dietary behaviour associated with obesity through online GMB workshops. By engaging with systems thinking and system dynamics, adolescents developed a comprehensive system map that effectively illustrated the complex interplay of factors influencing dietary behaviour. They identified critical risk factors such as pervasive marketing, the availability of unhealthy foods, and broader issues including mental health, body image, and eating disorders.

Adolescents proposed policy ideas aimed at addressing structural drivers beyond the food industry's direct influence, such as regulating social media influencers who promote unhealthy foods disguised as healthy. This reflects a recognition of the limitations faced by policymakers and public health practitioners, who often contend with limited local policy power and budgets compared to the food industry’s substantial resources.

The study underscores the importance of integrating adolescents' perspectives into policy development. Their insights and the identified feedback loops offer a foundation for creating targeted interventions and policies. By shifting the focus from individual behaviours to systemic drivers of unhealthy diets, this approach can enhance the effectiveness of public health strategies and better address the complexities of adolescent dietary behaviour.

## Supplementary Information

Supplementary Material 1.

## Data Availability

Personal data of the participants in this study and the video recording of the sessions and workshops cannot be made openly available because of ethical and legal considerations. Any data requests can be made directly to the lead author at yanaina.chavez-ugalde@mrc-epid.cam.ac.uk. Non-identifiable data supporting the results and conclusions of this article are included within the article. Non-identifiable data can be made available to bona-fide researchers on submission of a reasonable request to *datasharing@mrc-epid.cam.ac.uk*. The principles and processes for accessing and sharing data are outlined in the *MRC Epidemiology Unit Data Access & Data Sharing Policy*.

## Abbreviations

CLD: Causal Loop Diagram
GMB: Group Model Building
HFSS: High in Fat, Sugar, and Salt foods
SD: System dynamics
