# Supplementary material for: Using group model building to frame the commercial determinants of dietary behaviour in adolescence – findings from online system mapping workshops with adolescents, policymakers and public health practitioners in the Southwest of England
Source: BMC Public Health. 2025 Jan 14;25:144. doi: 10.1186/s12889-025-21320-7 (PMC11730463; doi:10.1186/s12889-025-21320-7)

**Supplementary material from Manuscript title:** “Using group model building to frame the commercial determinants of dietary behaviour in adolescence – Findings from online system mapping workshops with adolescents and policymakers and public health practitioners in the Southwest of England"

1. Outline of the three online GMB sessions with adolescents’


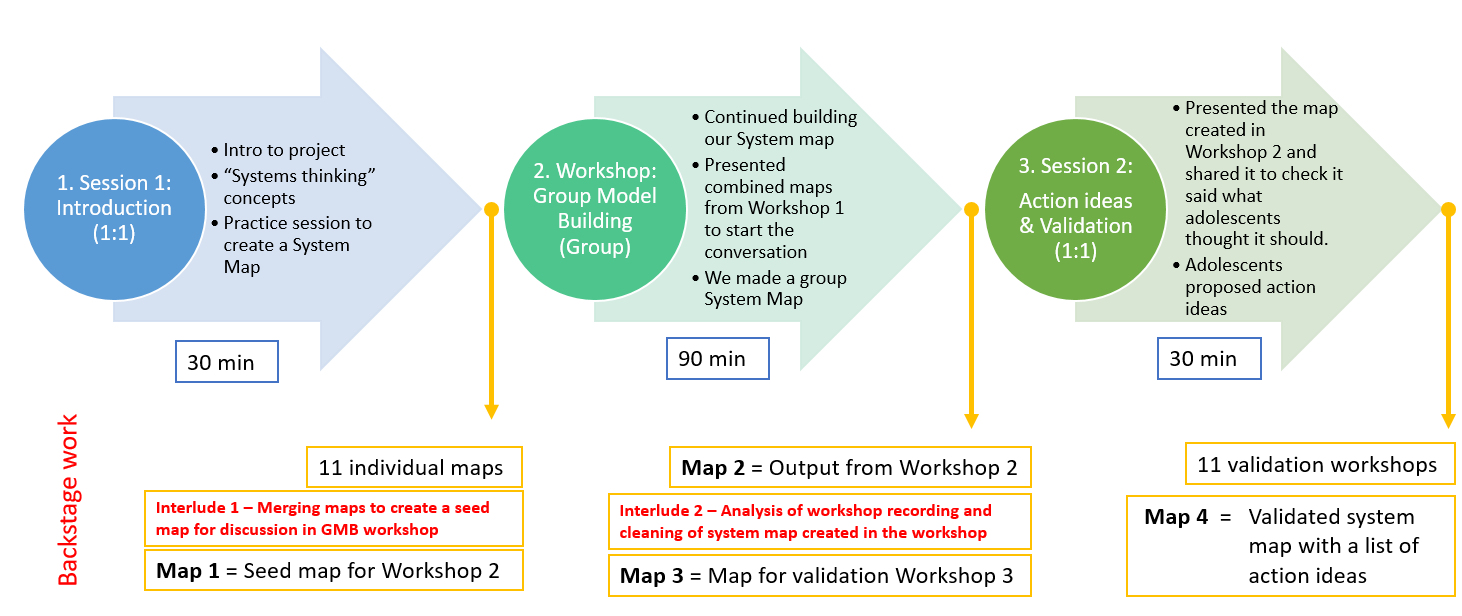

Supplement: Supplementary file 1 — Supplementary Material 1. [file 12889_2025_21320_MOESM1_ESM.docx]
